# Smoking cessation behavior in patients with a diagnosis of a non-communicable disease: The impact of perceived disease severity of and susceptibility to the disease

**DOI:** 10.18332/tid/170430

**Published:** 2023-10-06

**Authors:** Chie Taniguchi, Akihiko Narisada, Hirohiko Ando, Akane Hashimoto, Ayako Nakayama, Masaki Ito, Hideo Tanaka, Kohta Suzuki

**Affiliations:** 1 College of Nursing, Aichi Medical University, Nagakute, Japan; 2 Institute for Occupational Health Science, Aichi Medical University, Nagakute, Japan; 3 Department of Cardiology, Aichi Medical University, Nagakute, Japan; 4 Neyagawa City Public Health Center, Neyagawa, Japan; 5 Department of Health and Psychosocial Medicine, Aichi Medical University School of Medicine, Nagakute, Japan

**Keywords:** smoking cessation, non-communicable diseases, health belief model, perceived susceptibility, perceived severity

## Abstract

**INTRODUCTION:**

The Health Belief Model comprises two constructs influencing changed behaviors impacting on health, namely perceived severity and susceptibility. The aim of this study was to identify the impact of the combination of, or interactions between, these two constructs on quitting smoking in smokers with a diagnosis of a non-communicable disease (NCD).

**METHODS:**

From the large insurance claims database maintained by JMDC database (JMDC, Tokyo), we extracted data on 13284 participants who smoked. All participants were stratified according to their NCD diagnosis based on perceived severity and susceptibility as follows: Category I (high severity and high susceptibility) – acute myocardial infarction, and lung cancer; Category II (high severity and low susceptibility) – colorectal cancer, and stomach cancer; Category III (low severity and high susceptibility) – asthma, and transient ischemic attack; Category IV (low severity and low susceptibility) – appendicitis, and glaucoma. We performed multi-variable logistic regression analysis and calculated the proportion of those who were smoking at the first health check-up after the diagnosis and every three years thereafter.

**RESULTS:**

Using glaucoma as the reference, the adjusted odds ratios for smoking cessation were 14.2 (95% CI: 11.4–17.8) to 14.8 (95% CI: 12.5–17.4) in Category I; 4.5 (95% CI: 3.8–5.4) to 6.6 (95% CI: 5.4–8.0) in Category II; and 1.9 (95% CI: 1.7–2.1) to 2.8 (95% CI: 2.2–3.7) in Category III. In Categories I and II, the proportion of smokers rapidly decreased after diagnosis and mostly remained low thereafter. Smoking cessation rates for Categories I and II were not associated with readiness to improve lifestyles prior to NCD diagnosis.

**CONCLUSIONS:**

Our study confirms the significant impact of perceived severity of and susceptibility to the diagnosed disease on smoking cessation. The multiplicative effect of these two constructs at NCD diagnosis represents a ‘teachable moment’, a window of opportunity, for encouraging successful long-term smoking cessation.

## INTRODUCTION

Of all modifiable behaviors, smoking has the greatest impact on death from non-communicable diseases (NCDs)^1^. Continuous smoking increases the risk of NCD mortality, while 5 years after quitting smoking the risk is much reduced^2^. Therefore, it is important to stop smoking in order to maintain quality of life even after NCD diagnosis. The time of disease diagnosis is likely to represent a good opportunity for encouraging smoking cessation, i.e. it represents a teachable moment^3,4^. A literature review indicated that the best teachable moments for smoking cessation were the time of diagnosis of chronic disease, as well as at surgery and hospitalization following diagnosis^3^. Previous studies suggested that the diagnosis of NCDs initiates positive changes in multiple preventive behaviors including smoking cessation^5,6^.

The Health Belief Model is a theoretical framework that describes and explains the teachable moment and is commonly applied to understand and predict a wide range of health behaviors^7,8^. The model includes two constructs that influence health behaviors, namely, perceived severity of, and perceived susceptibility to, disease. The combination of these constructs results in a ‘perceived threat’ which motivates action. Perceived severity refers to the subjective assessment of the seriousness of a health problem and its potential consequences, such as the diagnosis of a deadly disease. Many studies have concluded that diagnoses of diseases which are generally serious or even fatal significantly increase the smoking cessation rate relative to diagnosis of a less serious disease^5,9,10^. In contrast, perceived susceptibility refers to a person’s subjective assessment of their risk of developing a health problem. Individuals who believe that they are susceptible to a particular health problem are more likely to engage in behaviors to reduce the risk of developing that health problem. People diagnosed with smoking-related diseases are more likely to quit smoking because of their perceived higher susceptibility due to smoking and the negative consequences for their health prognosis^11,12^.

A person’s recognition of perceived severity and perceived susceptibility is necessary for healthy behavior. However, the impact of the combination of these two constructs, or interactions between them, on smoking cessation behavior in smokers suffering from NCDs is still unclear. Thus, we investigated smoking cessation rates in NCD patients stratified according to the combination of perceived severity and perceived susceptibility in a large cohort study.

## METHODS

### Study design

This study was based on a real-world, longitudinal cohort study using a large claims database sourced from the Japan Medical Data Center Health Insurance Database (JMDC Inc., Tokyo, Japan).

### Setting

The JMDC claims-database covers >14 million employees and their families aged ≤74 years between January 2005 to 2022 in Japan. It contains individual-level anonymous medical claims data (e.g. inpatient, outpatient, medications) linked with annual health check-up data (e.g. anthropometric measurements, blood test results, and lifestyle data, such as alcohol consumption, smoking status, and receptiveness for advice on improving healthy lifestyles) according to the Industrial Safety and Health Act. Information on the International Classification of Diseases 10th revision (ICD-10) status is included in this database. Additionally, WHO-ATC codes were used for drug coding. This study was approved by the Institutional Review Board of Aichi Medical University, Japan (approval no.: 2020–082).

### Participants

Of a total of 4797329 insured people, we identified 1469239 individuals where claiming data could be confirmed at least once between January 2009 and December 2018. We classified the NCD diagnosis into four categories based on the following two criteria: 1) whether the diagnosed disease is generally considered serious and with a 5-year survival rate <75% (i.e. one quarter die within 5 years) (perceived severity); and 2) whether continuous smoking is associated with the progression and aggravation of the disease and whether or not smoking cessation after diagnosis is associated with improvements in disease outcomes (perceived susceptibility). Detailed criteria for the categorization of diseases are summarized in Table 1:

**Table 1:** Classification of NCD diagnoses in this study

*Perceived severity*	*Perceived susceptibility*	*Category*	*NCD diagnosis*	*ICD-10*	*Other extracted criteria*
High	High	I	AMI	I21	
			Lung cancer	C34	
High	Low	II	Colorectal cancer	C18 C19 C20	
			Stomach cancer	C16	
Low	High	III	Asthma	J45 J46	Those who were prescribed inhaled corticosteroid/long-acting β2-agonist (ICS/LABA) at least twice. ATC codes: R03AK06-07 R03AK10-11 R03AL R03BA01-02 R03BA05 R03BA07-09
			TIA	G459	
Low	Low	IV	Appendicitis	K35 K36 K37	Those with a history of hospitalization
			Glaucoma	H401 H406 H408 H409

- Category I (high perceived severity and high perceived susceptibility): acute myocardial infarction (AMI)^13,14^ and lung cancer^15,16^;
- Category II (high perceived severity and low perceived susceptibility): colorectal cancer^15^ and stomach cancer^15^;
- Category III (low perceived severity and high perceived susceptibility): asthma^17^ and transient ischemic attack (TIA)^18^; and
- Category IV (low perceived severity and low perceived susceptibility): appendicitis and glaucoma.

We extracted 1013991 records for those who were diagnosed with any of the above eight diseases and who had all been smokers at the time of their health check-up before the diagnosis.

**Figure 1:**
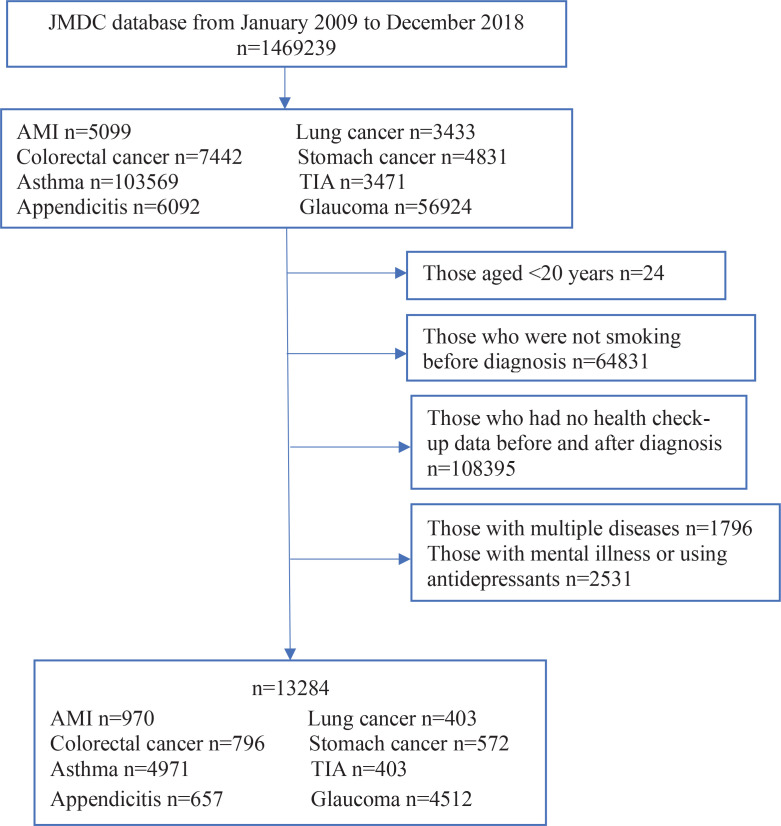
Flow chart of inclusion and exclusion criteria

Figure 1 shows a flow chart of the study inclusion criteria. We excluded suspected cases and those who had no health check-up data before and after diagnosis, and those aged <20 years who are forbidden to smoke by Japanese law. Additionally, we excluded patients with multiple diagnoses of these diseases, or who had a diagnosis of depression and were prescribed ≥2 of the following: selective serotonin reuptake inhibitors, serotonin and norepinephrine reuptake inhibitors, tricyclic antidepressants, or other antidepressant drugs (ATC codes: N06A4, N06A5, N06AA, N06A4, N06A5, N06AA, N05AX, N05AL). Finally, the records of 13284 patients were included in this study.

### Measures

The primary outcome of this study was to evaluate the smoking cessation rate within the first year after diagnosis. The secondary outcome was to compare changes in smoking rate every three years thereafter.

### Variables

We collected demographic and health check-up data including age, sex, and readiness to adopt healthier lifestyles at pre-diagnosis as classified into a: precontemplation stage, contemplation stage, and preparation stage/action stage, using Prochaskas’ transtheoretical model^19^. Readiness to adopt a healthier lifestyle was routinely assessed using self-administered questionnaires at the health check-up. Smoking status data (smoking/not smoking) were collected from annual health check-ups pre-diagnosis, within one year of diagnosis (first health check-up), and every three years thereafter. A smoking habit was defined as smoking >100 cigarettes over the entire lifetime, or smoking for >6 months and in the previous month. This definition is used by the Japanese health check-up questionnaire. Participants who answered ‘yes’ to a question about smoking habits were classified as smokers, whereas those who answered ‘no’ were classified as non-smokers. Relapse was defined as a smoker who once became a non-smoker and then changed back to a smoker.

### Statistical analysis

The summary statistics of participants were analyzed in four categories. To identify the impact of different diagnoses on smoking cessation, we performed multi-variable logistic regression analysis using glaucoma as the reference and adjusting for age and sex. The rate of smoking cessation accomplished was calculated for each category according to pre-diagnostic readiness for improving healthy lifestyles. To compare changes in smoking rate after diagnosis, we calculated the proportion of those who smoked at the first health check-up after the diagnosis and every three years thereafter. We also performed a sensitivity analysis for changes in smoking rate after diagnosis limited to those who participated at all follow-ups. Cochran-Armitage testing was performed to evaluate linear trends of unadjusted percentages.

Data analysis was performed with STATA ver.16 software (STATA Corp, College Station, TX). A p<0.05 (2-tailed) was considered statistically significant.

## RESULTS

### Baseline participant characteristics

The cohort comprised 11584 (87.6%) men, with 2126 (16%) aged <40 years, and 5822 (44%) aged ≥50 years. More than 40% of those participants diagnosed with asthma or appendicitis were in their 40s. More than half of the participants diagnosed with other diseases were aged ≥50 years (Table 2).

**Table 2:** Characteristics of the study subjects (N=13284)

*Characteristics*	*AMI*		*Lung cancer*		*Asthma*		*TIA*		*Colorectal cancer*		*Stomach cancer*		*Appendicitis*		*Glaucoma*	
	*n*	*%*	*n*	*%*	*n*	*%*	*n*	*%*	*n*	*%*	*n*	*%*	*n*	*%*	*n*	*%*
**Sex**																
Male	946	97.5	370	91.8	4124	83.0	356	88.3	721	90.6	540	94.4	600	91.3	3982	88.3
Female	24	2.5	33	8.2	847	17.0	47	11.7	75	9.4	32	5.6	57	8.7	530	11.8
**Age** (years)																
≤39	51	5.3	14	3.5	1361	27.4	21	5.2	54	6.8	15	2.6	178	27.1	440	9.8
40–49	341	35.2	132	32.8	2209	44.4	156	38.7	233	29.3	121	21.2	299	45.5	1811	40.1
≥50	578	59.5	257	63.7	1401	28.2	226	56.1	509	63.9	436	76.2	180	27.4	2261	50.1

### Smoking cessation rate at the first health check-up after diagnosis

Smoking cessation rates after diagnosis, in descending order, were Category I, II, III, and IV. The smoking cessation rate after diagnosis in Category I exceeded 59% (Table 3) whereas it ranged 31–40% in Category II. For Category III, the smoking cessation rate ranged 16–23%, and 9–13% for Category IV. The adjusted odds ratio for Category I was almost as great as multiplication for Categories II and III. Thus, the adjusted odds ratios for smoking cessation were 14.2 (95% CI: 11.4–17.8) to 14.8 (95% CI: 12.5–17.4) in Category I; 4.5 (95% CI: 3.8–5.4) to 6.6 (95% CI: 5.4–8.0) in Category II; and 1.9 (95% CI: 1.7–2.1) to 2.8 (95% CI: 2.2–3.7) in Category III (Table 3).

**Table 3:** Smoking cessation rates at the first health examination after diagnosis

*Category*	*Diagnosis*	*Total n*	*Smoking cessation rate %*	*AOR*	*95 % CI*
I	AMI	970	59.6	14.8	12.5–17.4
	Lung cancer	403	59.3	14.2	11.4–17.8
II	Colorectal cancer	796	31.8	4.5	3.8–5.4
	Stomach cancer	572	40.2	6.6	5.4–8.0
III	Asthma	4971	16.0	1.9	1.7–2.1
	TIA	403	22.6	2.8	2.2–3.7
IV	Appendicitis	657	12.6	1.5	1.1–1.9
	Glaucoma (Ref.)	4512	9.3	1	

AOR: adjusted odds ratio; multi-variable logistic regression analysis adjusting for age and sex.

### Trends in smoking cessation probability according to readiness to improve the lifestyle

Of the individuals whose diagnosis placed them in Category I, >50% of those who were precontemplating lifestyle improvement and smoking cessation before their diagnosis succeeded in quitting. Smoking cessation rates for those at the precontemplation stage, contemplation stage, and preparation or action stage before the diagnosis were 58.7%, 61.0% and 58.7%, respectively, for patients with an AMI diagnosis, and 65.2%, 54.0% and 59.9% for lung cancer patients (Table 4). In contrast, this was the case for <15% of those diagnosed with appendicitis and glaucoma (Category IV) or asthma (Category III). This rate significantly increased according to the position on the readiness scale for lifestyle improvement. Smoking cessation rates for patients at the precontemplation stage, contemplation stage, and preparation or action stage were 9.1%, 11.9% and 16.6%, respectively, for appendicitis patients, 6.9%, 9.8% and 10.5% for glaucoma patients, and 19.5%, 22.1% and 26.2% for asthma patients (p for trend: 0.022, 0.001, and 0.037, respectively) (Table 4). In contrast, participants whose NCDs were of perceived high severity (Category I and II) showed no significant increase in smoking cessation rates according to their level of readiness for lifestyle improvement.

**Table 4:** Trends in the post-diagnostic probability of quitting smoking according to pre-diagnostic readiness for improving healthy lifestyles (N=13284)

*Category*	*Diagnosis*	*Total*	*Precontemplation*		*Contemplation*		*Preparation/action*		*p for trend*
		*n*	*n*	*%*	*n*	*%*	*n*	*%*	
I	AMI	970	142	58.7	221	61.0	215	58.7	0.941
	Lung cancer	403	73	65.2	75	54.0	91	59.9	0.470
II	Colorectal cancer	796	59	26.7	105	34.1	89	33.3	0.134
	Stomach cancer	572	64	36.8	87	42.4	79	40.9	0.432
III	Asthma	4971	199	15.0	295	15.2	302	17.7	0.037
	TIA	403	24	19.5	34	22.1	33	26.2	0.207
IV	Appendicitis	657	18	9.1	29	11.9	36	16.6	0.022
	Glaucoma	4512	84	6.9	147	9.8	188	10.5	0.001

### Changes in smoking rate after diagnosis

**Figure 2:**
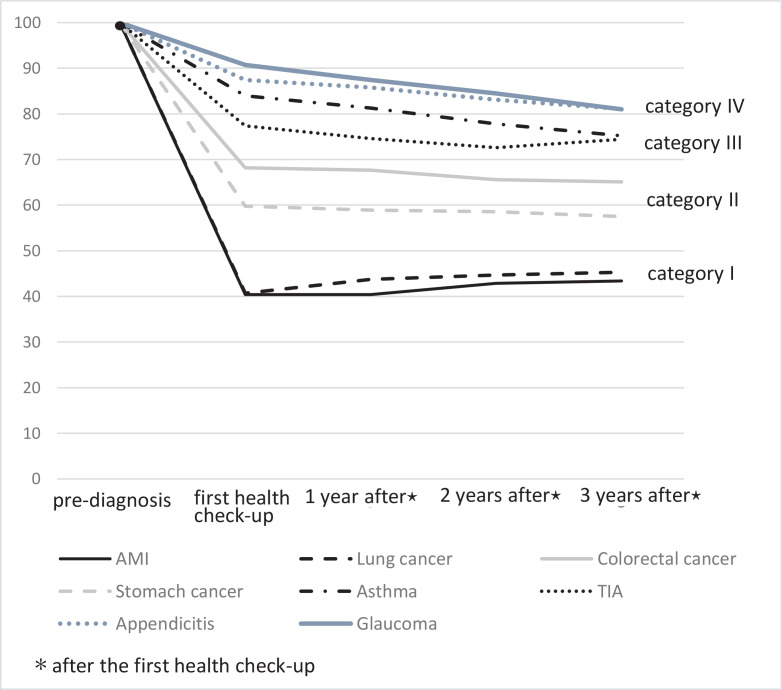
Changes in smoking rates after diagnosis: all participants

Figure 2 shows changes in smoking rate at the first health check-up and every three years thereafter. In Category I, the smoking rate after diagnosis mostly remained the same or had increased a little three years after the diagnosis (40.4% to 43.4% for AMI patients, 40.7% to 45.3% for lung cancer patients), neither being statistically significant (p for trend for AMI and lung cancer 0.20 and 0.24, respectively). Similarly, in Category II, smoking rates after diagnosis did not change in a linear manner. Percentages of patients continuing to smoke after a diagnosis of colorectal or stomach cancer were 68.2% before to 65.1% after (p for trend = 0.20), and 59.8% to 57.5% (p for trend = 0.52), respectively. On the other hand, in Category III and IV, almost 80% of participants had continued to smoke after their diagnosis, and smoking rates declined gradually thereafter. Percentages of patients continuing to smoke after a diagnosis of asthma and TIA were 84.0% to 75.2% (p for trend <0.01) and 77.4% to 74.4 % (p for trend = 0.248); for appendicitis and glaucoma these values were 87.4% to 81.1% (p for trend = 0.004) and 90.7% to 91.0% (p for trend <0.001); respectively.

We conducted sensitivity analyses limited to those who participated in all follow-ups (Supplementary file Figure). In Category I, almost 40% of participants continued to smoke after diagnosis, whereas in Category III and Category IV, this was almost 80%.

## DISCUSSION

Our analysis of smoking cessation rates in patients with any of eight NCDs supports the notion that perceived severity of, and perceived susceptibility to, a disease influences the likelihood of quitting smoking. The combination of high perceived severity and susceptibility defined by Category I diagnoses had the greatest impact on smoking cessation, and had a multiplicative effect in Category II and III patients. In addition, the smoking cessation rate for those with a high severity NCD diagnosis was not associated with their readiness to improve their lifestyle.

Individuals who perceived the severity of a disease are considered to recognize both the health consequences (life-threatening and disability or pain) and socioeconomic consequences (functioning at work, family life, economic issues) of the disease^20^. If the individual believes that the disease could have severe consequences for any aspect of their life, they will be more likely to change a health-relevant behavior^20^. Because the participants in our study were all of working age, it is plausible that many of them had such beliefs. This result indicated that participants who were diagnosed with serious NCDs did perceive severity not only for their health but also for detrimental impacts on their daily life, and this facilitated smoking cessation. Also, differences in smoking cessation rates depending on the diagnosis may be related to differences in the priority given to smoking cessation support by healthcare workers^3^. Perceived susceptibility to a given health condition depends on knowledge about that disease^20^. Healthcare workers need to provide knowledge of the benefits of smoking cessation on the state of the patient’s disease to increase their susceptibility to suggestions for improving lifestyles at the time of diagnosis^20^. Furthermore, it is important to convince the patient that smoking cessation is personally relevant, being as specific as possible^21^.

The smoking cessation rate for those with a high severity NCD diagnosis was not associated with their readiness to improve their lifestyle. Even when the patients were at the precontemplation stage, the smoking cessation rate was no different from those who were already at the preparation stage. This indicates that many participants rapidly changed their behavior on learning of a serious diagnosis, even when they had been in a low state of readiness for improving their lifestyle before that. Consistent with this, the transtheoretical model shows that when the stage is raised, it becomes easier to take action due to the occurrence of emotional experiences related to health behavior even in someone at the precontemplation stage^22^. Smoking rates in those with high severity NCD diagnoses were mostly unchanged >2–3 years after diagnosis. Generally, as in populations with low perceived severity, readiness for lifestyle improvement, especially smoking cessation, increases over time. Due to this increasing readiness, smoking rates gradually decrease. In contrast, regardless of their readiness to improve their lifestyle, a diagnosis of higher severity disease was associated with a higher proportion of smoking cessation immediately after the diagnosis.

### Strengths and limitations

A strength of this study is that it is longitudinal, using a large sample consisting of insurance records from the Japanese population, thus allowing for sensitivity analyses that strengthen the robustness of the results. Another strength is that because our study using real-world data included a very large number of people, we were able to evaluate outcomes according to different NCD diagnoses. Our study also has some limitations. First, we used medical claims data. ICD-10 codes were used for all diagnoses. We excluded suspected cases, but there may be differences in the recorded ICD-10 codes and actual diagnoses. Second, we did not directly assess perceived severity or perceived susceptibility at NCD diagnosis at the individual level but defined these items based on the NCD diagnosis. This might increase misclassification in the four categories, which would underestimate the differences of smoking cessation rates among the categories. Third, our study had limited representability. It included only health check-up data of Japanese employees and all study participants were aged <75 years. Additionally, due to differences in medical technology and perceptions of diseases diagnosis, further studies are needed in different countries. Fourth, other potential confounders such as smoking-related factors (number of cigarettes smoked per day, duration of current smoking, and nicotine dependence score) and socio-economic factors should ideally have been considered. Nevertheless, our primary aims were to compare the smoking cessation rates after diagnosis of several different diseases, so the impact of this limitation is likely to be small because diagnosis of disease would include these potential factors.

## CONCLUSIONS

Our study suggests that perceived severity and susceptibility had a strong impact on smoking cessation after NCD diagnosis, and that these two items had multiplicative effects in this context. It is important for healthcare workers at this unique teachable moment to encourage the patient to understand why smoking cessation is personally relevant, being as specific as possible on benefits regarding disease progression.

## Data Availability

The data supporting this research cannot be made available for privacy or other reasons. The dataset that was used is held by the Japan Medical Data Center (JMDC) (https://www.jmdc.co.jp/en/). Access to the data for research is available after filing a formal data access request with https://www.jmdc.co.jp/en/inquiry. Requestors need to accept the terms and conditions of the data request and may need to pay the corresponding data access fee.
