# Supplementary material for: Smoking cessation behavior in patients with a diagnosis of a non-communicable disease: The impact of perceived disease severity of and susceptibility to the disease
Source: Tob Induc Dis. 2023 Oct 6;21:125. doi: 10.18332/tid/170430 (PMC10557053; doi:10.18332/tid/170430)
Supplement: Supplementary file 1 [file TID-21-125-s1.pdf]

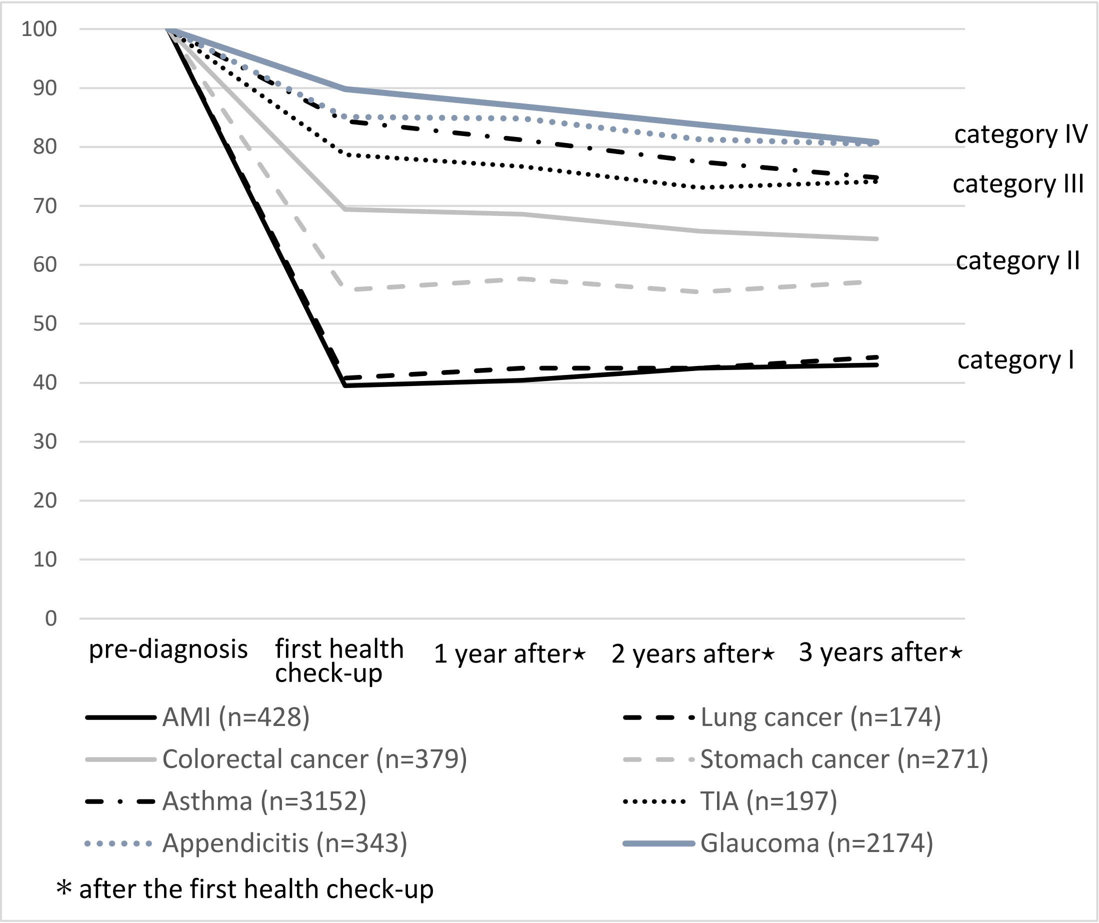

Appendix figure. Changes in smoking rate after diagnosis limited to those who had smoking status recorded at all follow-ups (n=8,393).

© 2023 Taniguchi C. et al.
